# Parallel imaging of *Drosophila* embryos for quantitative analysis of genetic perturbations of the Ras pathway

**DOI:** 10.1242/dmm.030163

**Published:** 2017-07-01

**Authors:** Yogesh Goyal, Thomas J. Levario, Henry H. Mattingly, Susan Holmes, Stanislav Y. Shvartsman, Hang Lu

**Affiliations:** 1 Department of Chemical and Biological Engineering, Princeton University, Princeton, NJ 08544, USA; 2 Lewis-Sigler Institute for Integrative Genomics, Princeton University, Princeton, NJ 08544, USA; 3 School of Chemical and Biomolecular Engineering, Georgia Institute of Technology, Atlanta, GA 30332, USA; 4 Department of Statistics, Stanford University, Stanford, CA 94305, USA

**Keywords:** Microfluidics, Ras, Mutations, Capicua, SVD

## Abstract

The Ras pathway patterns the poles of the *Drosophila* embryo by downregulating the levels and activity of a DNA-binding transcriptional repressor Capicua (Cic). We demonstrate that the spatiotemporal pattern of Cic during this signaling event can be harnessed for functional studies of mutations in the Ras pathway in human diseases. Our approach relies on a new microfluidic device that enables parallel imaging of Cic dynamics in dozens of live embryos. We found that although the pattern of Cic in early embryos is complex, it can be accurately approximated by a product of one spatial profile and one time-dependent amplitude. Analysis of these functions of space and time alone reveals the differential effects of mutations within the Ras pathway. Given the highly conserved nature of Ras-dependent control of Cic, our approach provides new opportunities for functional analysis of multiple sequence variants from developmental abnormalities and cancers.

## Abstract (precis)

**Summary:** Microfluidics, live imaging and systems biology techniques are combined to develop a new approach for the functional analysis of sequence variants in the highly conserved Ras signaling pathway.

## INTRODUCTION

The Ras signaling pathway is a highly conserved regulator of adult and developing animal tissues. Studies in model organisms were essential in delineating this critical pathway via genetic screens that relied on analyses of the morphological defects in specific Ras-dependent structures, such as the insect eye or nematode vulva (Brunner et al., 1994; Freeman, 1996; Han and Sternberg, 1990; Han et al., 1993; Kornfeld et al., 1995; O'Neill et al., 1994; Schupbach and Wieschaus, 1986; Sternberg and Han, 1998). One of the main advantages of these screens, which continue to reveal new components and interactions, is their ability to examine large numbers of samples, providing the basis for follow-up studies at molecular and cellular levels. With the advent of live imaging, it became possible to not only analyze the ultimate morphological outcomes of genetic perturbations, but also to monitor the dynamics of signaling. However, most live-imaging studies are limited in the number of organisms that can be examined at any given time, which is in contrast to the high-throughput nature of earlier work in model genetic systems. Here, we present an experimental and analytical strategy that addresses this problem, providing new opportunities for the quantitative analysis of the Ras pathway and its genetic perturbations.

**Fig. 1.**
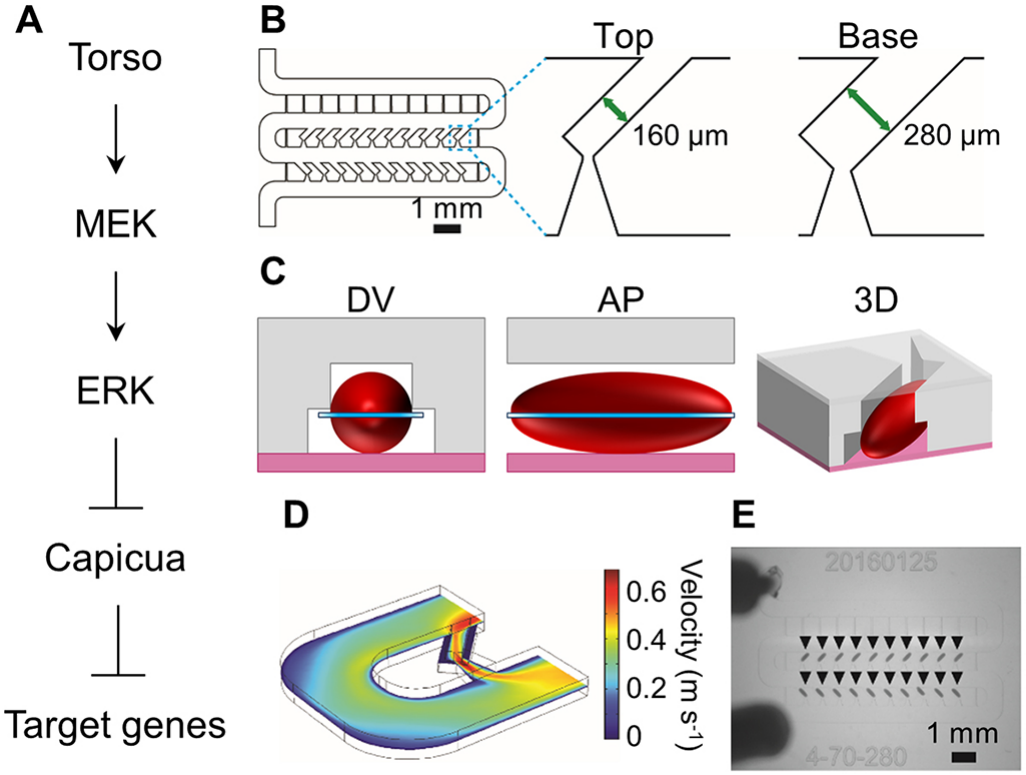
**Microfluidic array for parallel imaging of live embryos.** (A) Torso/ERK signaling antagonizes Cic-dependent gene repression. (B) Schematic of the microfluidic array with close-up views of a single-trapping unit in both layers. (C) Illustrations depicting a single embryo (red) within a trapping unit; dorsal-ventral (DV) and anterior-posterior (AP) cross-sections, and three-dimensional (3D) representations are depicted. (D) Superficial fluid velocity at the mid-plane of the trapping unit predicted by the finite element model. (E) Representative image of a loaded microfluidic device: black triangles indicate embryos trapped within the array.

Our strategy is based on parallel imaging of the early *Drosophila* embryo, a system that combines low cost, simple anatomy, and amenability to a wide range of genetic manipulations. We focus on Ras signaling during the time when the embryonic termini are patterned by a locally activated receptor tyrosine kinase (RTK), Torso (Casanova and Struhl, 1989). Torso is active only at the poles, reflecting the localized processing of its secreted ligand (Furriols and Casanova, 2003). Signaling through the Ras pathway, Torso induces the dual phosphorylation and activation of the Extracellular signal-regulated kinase (ERK), an enzyme with multiple intracellular substrates (Fig. 1A) (Gabay et al., 1997). One of the main substrates of ERK in the early embryo is a DNA-binding transcriptional repressor Capicua (Cic), which acts as a common sensor of the Ras pathway in adult and embryonic tissues (Futran et al., 2013; Jiménez et al., 2000; Jiménez et al., 2012; Lee et al., 2002; Liao et al., 2017; Okimoto et al., 2016). In response to direct phosphorylation by ERK, Cic is exported from the nucleus and degraded in the cytoplasm, enabling the expression of genes needed for the formation of nonsegmented terminal structures in the future larva (Grimm et al., 2012; Johnson et al., 2017; Lim et al., 2013).

The spatial pattern of Cic in the early embryo is very sensitive to Ras activation, suggesting that it could be used to develop a strategy for quantitative comparison of mutations associated with deregulated Ras signaling. However, this pattern is very dynamic, reflecting the fact that Ras-dependent control of Cic coincides with nuclear divisions. Therefore, quantitative comparison of Cic patterns requires live imaging. Until recently, live imaging of Cic has been possible only in one embryo at a time (Grimm et al., 2012). We have designed a microfluidic device with new features for parallel imaging of Cic dynamics in dozens of embryos and demonstrate that this enables quantitative analysis of the differential effects of mutations within the Ras pathway. Given the highly conserved nature of the signaling cascade that leads from Torso activation to Cic downregulation, our approach can be used for functional analysis of the rapidly accumulating Ras pathway mutations found in human diseases (Hanahan and Weinberg, 2011; Jindal et al., 2015; Rauen, 2013).

## RESULTS

### Microfluidic device for parallel imaging of Cic dynamics in embryos

The spatiotemporal pattern of Cic in the early embryo reflects the combined effects of spatially uniform translation of Cic from maternally deposited transcript, localized control of Cic nuclear localization and degradation, and ongoing nuclear division. Ras-dependent ERK activation at the poles increases the rate of Cic nuclear export and decreases nuclear import. At the same time, the levels of Cic are high and largely nuclear in the middle of the embryo, where the Ras pathway is inactive. All of this happens when the embryo is still a syncytium, where nuclei are dividing in a shared cytoplasm. At the onset of every interphase, the nuclear levels of Cic are essentially zero, but they are rapidly re-established. A combination of these processes results in a dynamic pattern where the levels of Cic remain low at the poles, increase in the middle of the embryo and are punctuated by nuclear divisions (Grimm et al., 2012).

Given these complex dynamics, quantitative comparisons of Cic patterns across mutant backgrounds requires data from multiple embryos, ideally imaged under the same conditions. We developed a microfluidic chip optimized for this specific purpose. As a starting point, we used our earlier design of a microfluidic array that orients embryos in the upright position (Chung et al., 2011; Levario et al., 2013; Levario et al., 2016a,b). Since Cic protein in the early embryo is distributed along the anteroposterior axis, we redesigned our array to laterally orient embryos (Fig. S1). The new design has a two-step geometry with a wide base and a narrow top (Fig. 1B). The wide base enables aberration-free imaging near the midsagittal section of the embryo (Fig. 1C, Fig. S2).

To optimize the embryo-trapping efficiency in the array, we used finite element calculations to tune the fluid resistances in the main flow manifold and trap channels. The trapping efficiency in the new array is greater than 90%, which enables data collection from dozens of embryos in a single imaging experiment (Fig. 1D,E). After confirming that the trapped embryos develop normally in our microfluidic array, we used it to monitor Cic levels starting from nuclear cycle 10 to gastrulation. Consistent with previously established results (Grimm et al., 2012), we found Cic levels to be low at the poles and elevated toward the embryo mid-body, wherein Cic levels are uniform throughout the central ∼50% of the anterior-posterior axis.

### Low-dimensional approximation of spatiotemporal dynamics

**Fig. 2.**
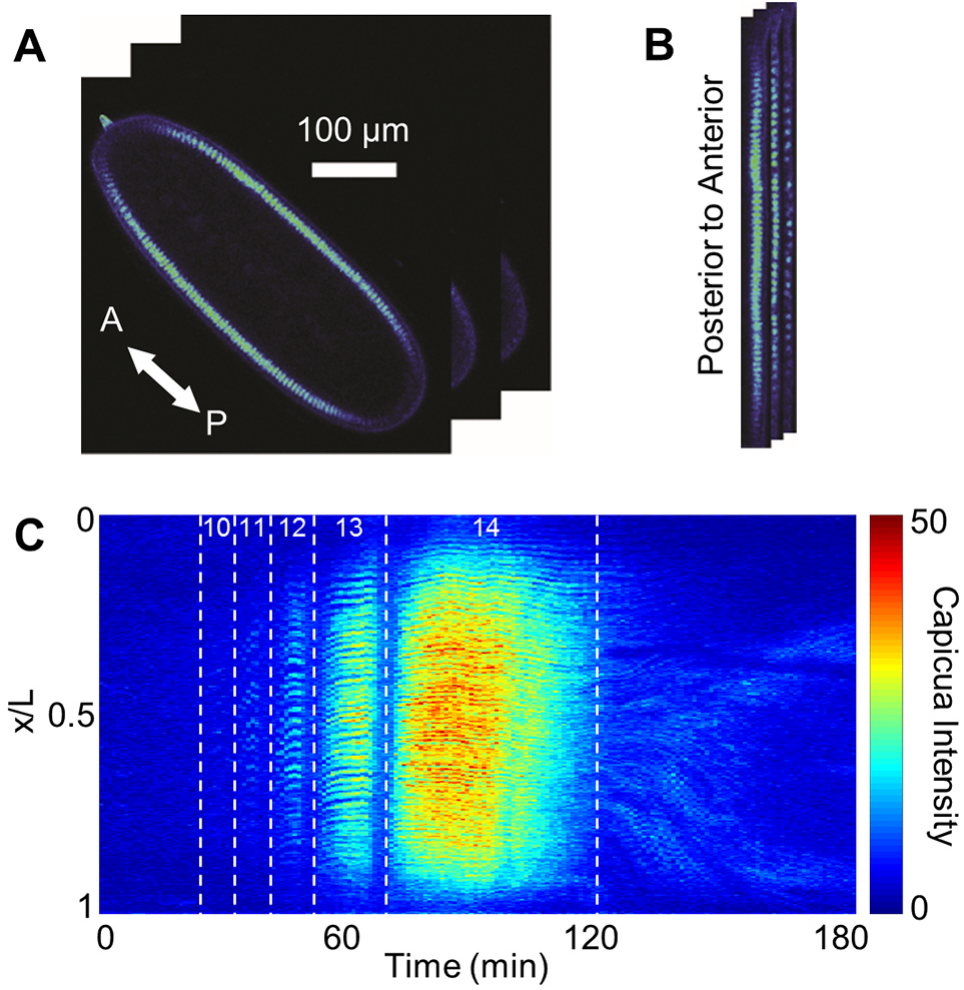
**Image processing of time-lapse data of Cic dynamics.** (A) Time-lapse images of a representative Capicua-Venus (CicV)-expressing embryo. (B) Time-lapse images of a single optical cross-section the blastoderm embryo. (C) Representative space-time plot of Cic dynamics. Vertical axis represents egg length (L) from anterior to posterior (0-1), horizontal axis represents imaging time, and color axis represents CicV intensity (arbitrary units). White vertical dotted lines indicate nuclear cycle transitions with cycles labeled for stages 4 and 5 of embryogenesis. Time is set to zero at the start of imaging.

To analyze the large amount of data acquired in these experiments, we developed a pipeline for image processing and data representation. First, the raw live-imaging data from the midsagittal optical sections of individual embryos were converted into a two-dimensional matrix, in which the columns are the anteroposterior profiles of Cic at consecutive time points (Fig. 2). This matrix serves as a starting point for analyzing the spatiotemporal pattern of Cic across the embryo. In addition, we used image segmentation to extract the temporal dynamics of Cic levels from individual nuclei (Fig. S3). Thus, our approach provides access to Cic dynamics on the scales of both a single cell and the whole tissue.

**Fig. 3.**
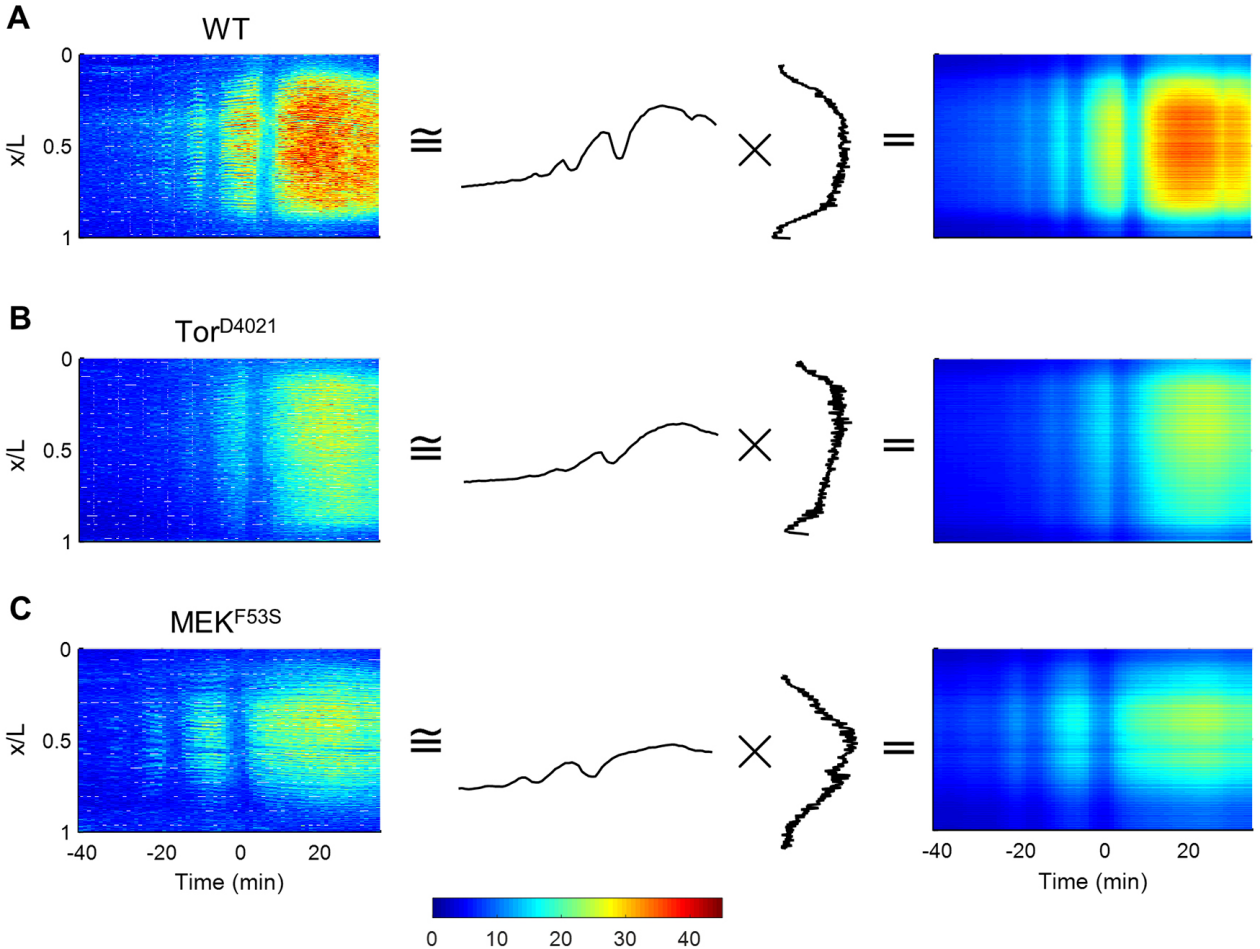
**Low-dimensional approximation of Cic dynamics.** (A-C) Reconstruction of Cic dynamics using singular value decomposition for WT (A), Tor^D4021^ (B), MEK^F53S^ (C). Single embryo raw data (left column), associated first spatial mode and projection (middle column) and reconstructed heat maps (right column). Time is set to zero at the 13th mitotic division. Color scale represents CicV intensity (arbitrary units).

As discussed above, the dynamic pattern of Cic arises as a combination of processes that depend on time, such as the spatially uniform nuclear divisions in the blastoderm, and on space, such as Ras signaling at the poles. We hypothesized that the spatiotemporal pattern of Cic could be approximated by a separable function – that is, a product of two functions, one of which depends only on time and the other depends only on space. To test this idea, we used the singular value decomposition (SVD) to find rank-one approximations for the matrices describing the spatiotemporal patterns of Cic extracted from live imaging data. This approximation is given by the product of the left and right singular vectors corresponding to the leading singular value. Fig. 3A shows that these functions reveal the spatial structure of the Cic pattern (high in the middle of the embryo, low at the poles) and its temporal dynamics (increasing levels, punctuated by sequential mitoses). We defined a measure of the accuracy of the rank-one approximation based on the singular values that equals one when the approximation is exact (see Materials and Methods). Our analysis of the Cic space-time matrices revealed that the accuracy measures were greater than 0.96 for all embryos, indicating the appropriateness of the rank-one approximations.

Finally, we tested whether this representation of Cic dynamics can also be used in multiple genetic backgrounds. Towards this end, we collected ensembles of spatiotemporal data from embryos with genetic perturbations of Ras signaling (see below). In all cases, we found that rank-one approximations were accurate (>0.95, based on the defined measure). Representative examples are shown in Fig. 3B,C. Visual inspection of the leading spatial profiles and their amplitudes reveals that different backgrounds can be compared based on the quantitative properties of these one-dimensional functions. This is the basis for our statistical analysis in the next section.

### Statistical comparison across genetic backgrounds

To test whether the presented approach can be used to quantitatively compare the effects of genetic perturbations, we analyzed Cic dynamics in embryos from three different genetic backgrounds. The first set of embryos carried a dominant mutation in the gene encoding Torso (*tor*). This mutation was discovered in a mutagenesis screen and results in ligand-independent activation of Ras signaling, leading to major defects in embryo segmentation (Klingler et al., 1988). Similar patterning defects (progressive loss of segments) can be caused by misexpression of activating variants of Dsor1, a *Drosophila* ortholog of human MEK1 (also known as MAP2K1), which is a critical component of the Ras pathway (Goyal et al., 2017). Wild-type MEK1 (and Dsor1) is activated only in response to extracellular ligands. However, MEK1 activity can become ligand independent for several point mutations found in human diseases, including developmental abnormalities and cancers (Goyal et al., 2017; Jindal et al., 2017). Here, we analyzed the effects of F53S and E203K mutations in MEK1, which are associated with Cardio-facio-cutaneous syndrome and melanoma, respectively (Bromberg-White et al., 2012).

**Fig. 4.**
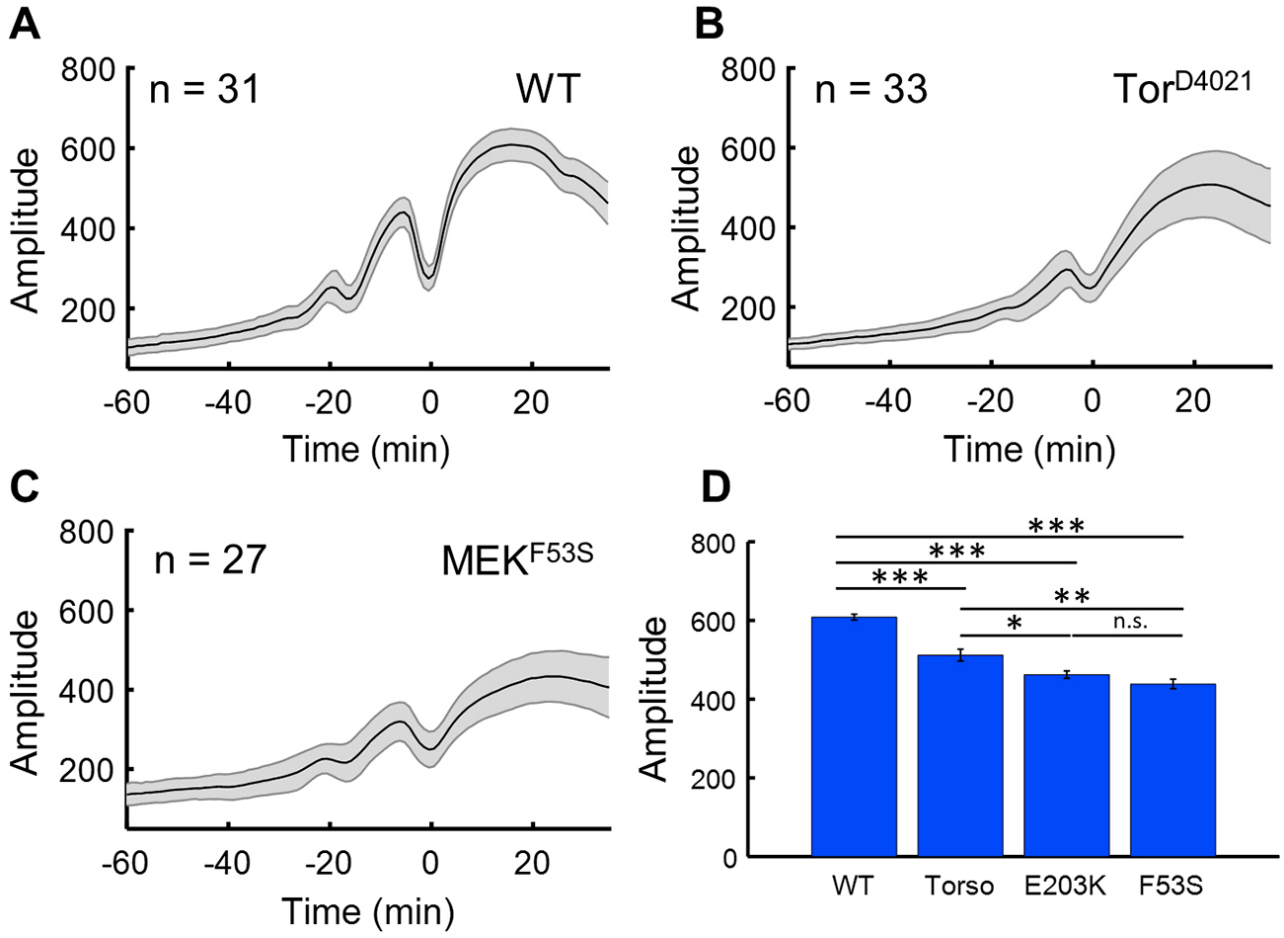
**Statistical analysis of Cic dynamics in wild-type and mutant embryos.** (A-C) Dynamics of the amplitudes of the first spatial mode for the wild type and for Tor^D4021^ and MEK^F53S^ mutants. (D) Comparison of mean amplitude in cycle 14 for each genotype reveals significant pairwise differences between genotypes, except for the MEK mutants. Throughout the figure, the shaded regions indicate one standard deviation. Student's *t*-test, two-tailed: *P<0.05, **P<0.005, ***P<0.0005; n.s., not significant.

**Fig. 5.**
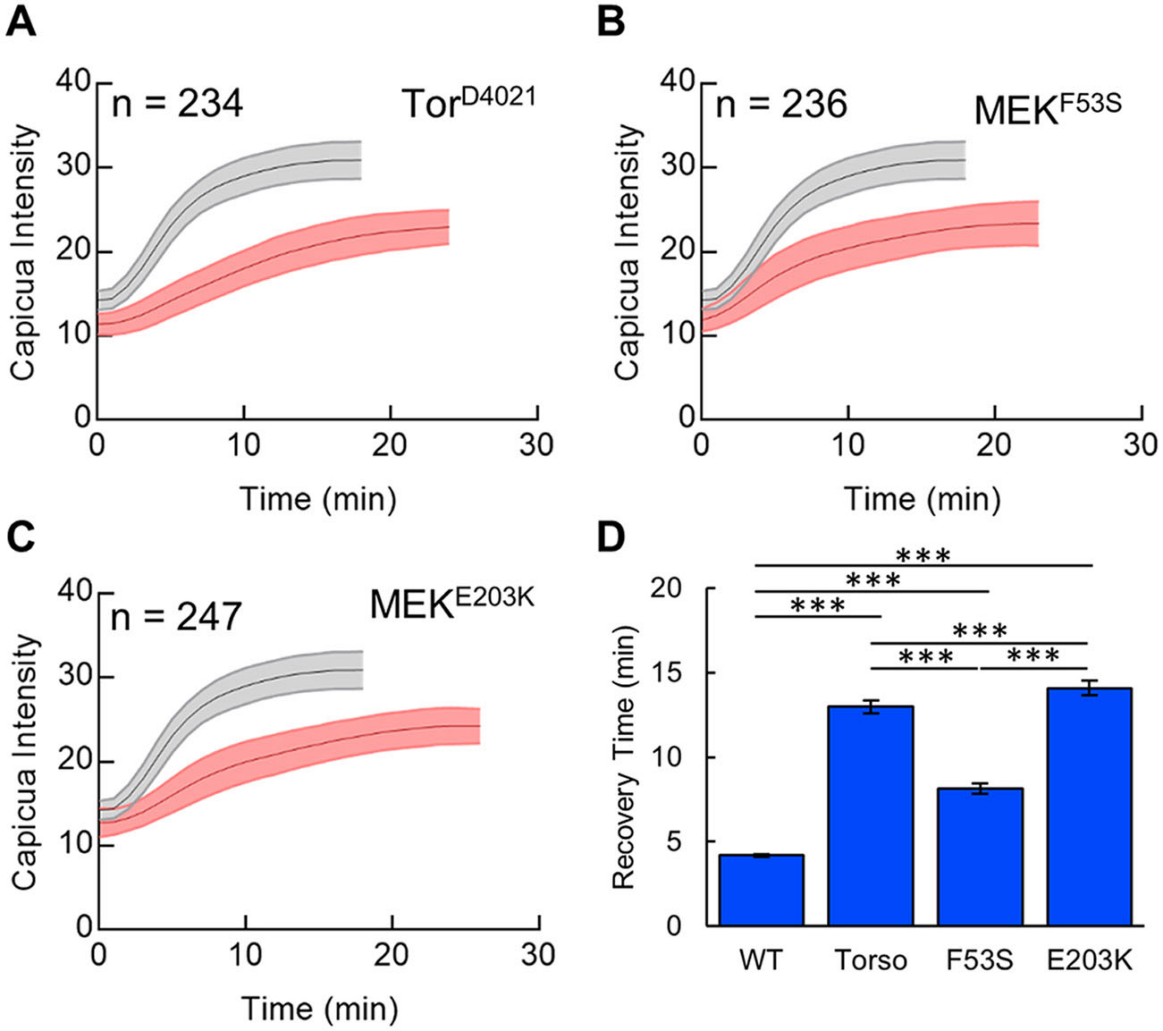
**Single-cell analysis of the Cic import rates.** (A-C) Mean single-cell CicV intensity traces for the wild type and Tor^D4021^ (A), MEK^F53S^ (B) and MEK^E203K^ (C) mutants. The wild type results are shown in gray in each plot. (D) Comparison of recovery times reveals significant pairwise differences between genotypes. Throughout the figure, the shaded regions and error bars indicate one standard deviation. Student's *t*-test, two-tailed: **P<0.005, ***P<0.0005; n.s., not significant.

Since each of these genetic perturbations results in ligand-independent activation of ERK, we expected to see a significant reduction of Cic levels in the middle of the embryo, where the Ras pathway is normally inactive. Accordingly, we found that the maximal amplitude of Cic at nuclear cycle 14 for mutants was significantly lower than that in the wild type (Fig. 4). Furthermore, recent studies in zebrafish suggest that the intrinsic strength of the F53S mutation should be less than that of the E203K mutation (Jindal et al., 2017). No difference in the amplitudes of the tissue-level profiles was detected (Fig. 4D). However, a difference became clear when we analyzed the recovery rates of the nuclear Cic levels after each mitosis (Fig. 5A-C, Fig. S4). Specifically, the recovery of nuclear Cic levels was slowed in embryos expressing the E203K sequence variant of Dsor1 compared with the F53S mutation (Fig. 5D). Since nuclear import rates of Cic depend on the levels of ERK activation, this metric suggests that E203K leads to stronger ERK activation than the F53S mutation.

**Fig. 6.**
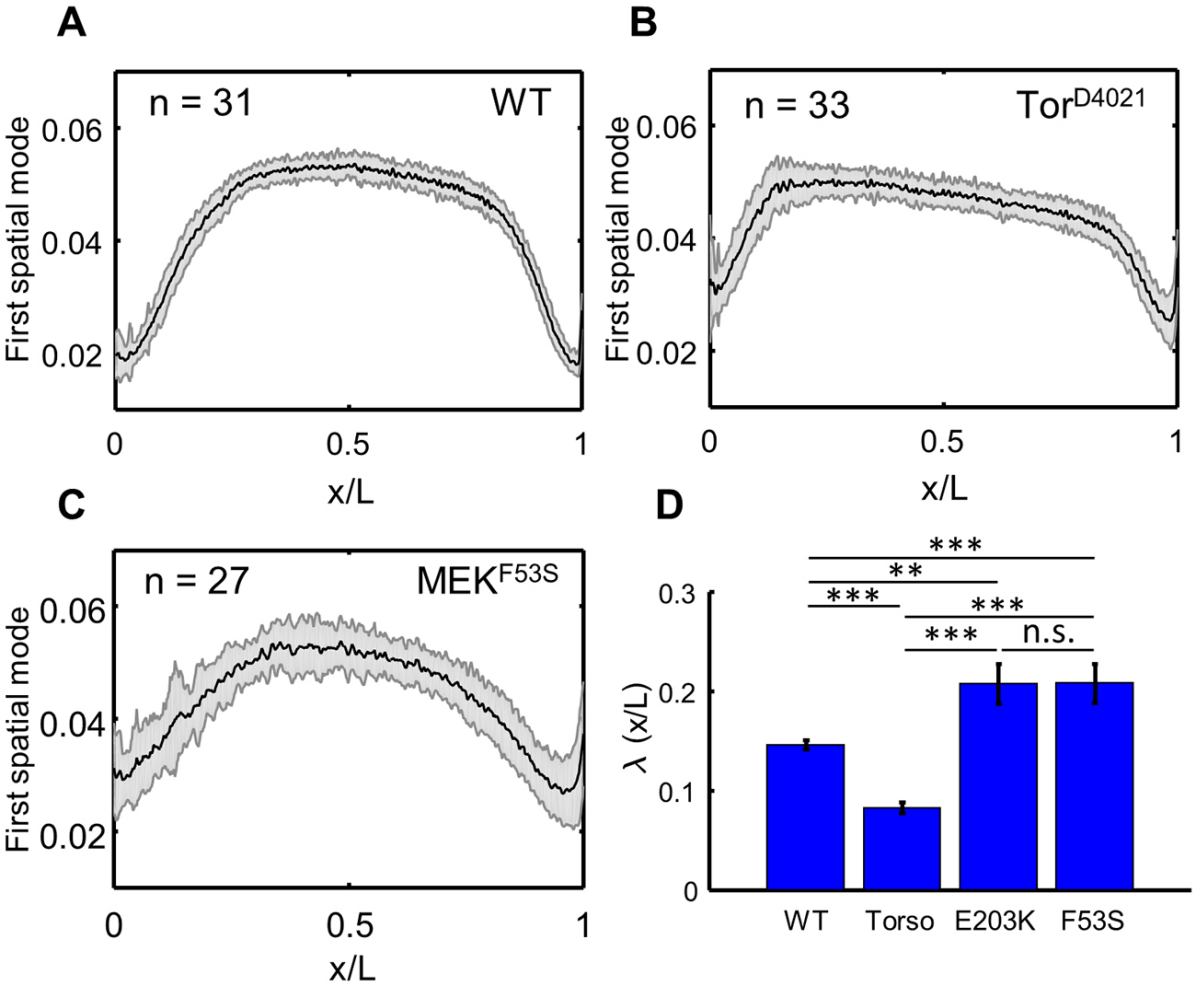
**Statistical comparison of spatial profiles.** (A-C) Profiles of the dominant modes for the wild type (A) and Tor^D4021^ (B), and MEK^F53S^ (C) mutants across the embryo length (L). (D) Pairwise comparison of characteristic length, λ. The gray shaded regions indicate one standard deviation. Student's *t*-test, two-tailed: **P<0.005, ***P<0.0005; n.s., not significant.

Based on the low-dimensional approximation of the Cic pattern, it appeared that the spatial profile of Ras signaling depends on the nature of the genetic perturbation. Specifically, embryos expressing the activating variants of MEK1 had a graded Cic profile, as opposed to the sharper pattern observed in embryos with constitutively active Torso (Fig. 6A-C). We quantified this by fitting the anterior 40% of each of the first spatial modes with a Hill function and compared the position of half-maximal accumulation of Cic, denoted λ (Fig. 6D). Although the origins of these differences are unclear at this point, we speculate that they reflect differences in the spatial localization of the affected components of the signaling pathway. The Torso protein is confined to the membrane, where the rates of diffusion are low, whereas Dsor1 localizes to the cytoplasm, where proteins have higher mobility. Taken together, our approach can be used to compare differential strengths of multiple genetic perturbations of Ras signaling.

## DISCUSSION

The highly conserved nature of the Ras pathway suggests that functional studies of mutations from human diseases can be carried out in *Drosophila*, capitalizing on the reproducible nature of several well-characterized Ras-dependent developmental signaling transients. Our approach relies on approximating the spatiotemporal pattern of Cic as a product of one dominant spatial mode and one time-dependent amplitude, using data from many embryos that do not have to have exact age synchronization. Thus, complex signaling transients in different mutants could be compared based on the differences of these functions of space and time alone. We demonstrated that statistical analysis of these functions can be used to detect the quantitative differences between the effects of mutations at different levels of the pathway. Based on this, we propose that our live-imaging framework can be used to evaluate the functional consequences of many sequence variants identified in human developmental abnormalities and cancers. Indeed, since most of the sequence variants associated with human diseases occur in conserved positions of the signaling proteins, their effects can be readily assessed in *Drosophila*.

One of the first questions associated with evaluating the newly discovered sequence variants is whether they cause activation of the ligand-independent pathway. The early *Drosophila* embryo provides an ideal system for answering this question for the Ras pathway components. Besides confirming via reduced Cic amplitude in mutant backgrounds that they are indeed intrinsically active, our tissue-level and single-cell assays can differentiate between their strengths and properties.

## MATERIALS AND METHODS

### Fabrication of the two-layer anterior-posterior array

A previously developed method of rapid prototyping was employed in this study to fabricate PDMS microfluidic devices. This method involved a combination of master mold microfabrication, and PDMS replica molding described below.

### Master mold fabrication and PDMS replica molding

Master mold fabrication involved a two-layer, photolithographic procedure to pattern two distinct layers with two different photomasks. Two photomasks were designed in AutoCAD (AutoDesk), and printed onto high-resolution transparencies (CAD Art Services) in order to be used during pattern transfer. Each photomask layer was designed to be 100 µm tall for a total device height of 200 µm. To achieve the desired layer heights, we employed the thick photoresist SU-8 2100 (MicroChem), and used a specifically tuned lithographic process. Silicon wafers were dehydrated by baking at 150°C for 30 min. The wafer was allowed to cool to room temperature after each bake step before proceeding to the next step. A 100-µm-thick layer of SU-8 2100 photoresist was deposited by a two-step spinning protocol with the following parameters: (1) 100 rpm s^−1^ acceleration, 500 rpm spin speed and 10 s spin duration; (2) 300 rpm s^−1^ acceleration, 3000 rpm spin speed and 30 s spin duration. The resist film was dried by baking at 65°C for 5 min and 95°C for 20 min. The photoresist was patterned with the first layer photomask by exposing the film to 365 nm wavelength light with an exposure energy of 240 mJ cm^−2^. Resist crosslinking was expedited by baking at 65°C for 5 min and 95°C for 10 min. The previous steps, beginning at photoresist deposition, were repeated in order to deposit the second layer of photoresist and pattern with the second layer photomask. The uncross-linked resist was removed by soaking it in an agitated bath of SU-8 developer (propylene glycol methyl ether acetate; Sigma Aldrich). The mold was then rinsed with isopropyl alcohol, and allowed to air dry. Finally, the mold was exposed to silane vapor [(tridecafluoro-1,1,2,2-tetrahydrooctyl)-1-trichlorosilane; United Chemical Technologies] to facilitate casting ejection during subsequent replica mold processing.

PDMS replica mold processing started by pouring and curing two distinct layers of PDMS pre-polymer onto the master mold that was constructed in the previous paragraph. The first layer of PDMS (Dow Corning) was mixed with a 15:1 monomer:crosslinker ratio, and poured to ∼1 mm thick onto the master mold surface. The first PDMS layer was partially cured for 30 min at 75°C. A second layer of PDMS was mixed with a 7.5:1 monomer:crosslinker ratio, poured to ∼5 mm thick onto the partially cured first layer of PDMS, and placed back in the oven at 75°C and allowed to cure for an additional 2 h. The softer base layer allowed the array features to be more flexible, while the harder top layer makes the overall mold more rigid for easy plasma bonding in the next steps. PDMS replica molds were ejected from SU-8 master molds, diced, and pierced with 19 g blunt-tip needles to create fluidic access ports. Finally, PDMS molds were treated with oxygen plasma and allowed to contact glass coverslips to form fully enclosed microfluidic devices.

### Embryo preparation for loading into the microfluidic array

Adult flies were placed on a fresh apple juice agar plate and allowed to deposit eggs for 1 h and 15 min. Embryos were subsequently dechorionated by bathing embryos in a gently agitated bleach solution of 2.5% sodium hypochlorite for 1.5 min. Eggs were rinsed with 10 ml deionized water and suspended in 1 ml of 0.15% Triton X-100 surfactant in phosphate-buffered saline (PBST) in an Eppendorf tube. Embryos were rinsed three times with filtered PBST solution to remove unwanted particulates from the egg suspension including, partially dissolved chorion membranes. This step is especially important for microfluidic arraying, because these particulates can also be trapped by the array and introduce optical aberrations during imaging.

### Three-dimensional finite element modeling of the trapping array

Finite element modeling was performed with COMSOL Multiphysics 4.3b Modeling Software. For simplification, a single trapping unit was modeled consisting of an embryo trap, resistance channel and one turn of the main serpentine channel. The three-dimensional (3D) steady-state incompressible Navier-Stokes equations were solved to visualize flow through the traps. For boundary conditions, the inlet superficial velocity that was experimentally estimated to be ∼0.1 m/s, an assumed outlet condition of atmospheric pressure and no slip along channel surfaces.

### Stocks and fly husbandry

Capicua-Venus homozygous (*CicV/CicV*) stocks used in step 1 were crossed with OregonR to generate heterozygous (*CicV*/+) embryos for analysis. Males from *Tor^D4021^/CyO* were crossed with females from *CicV/CicV* to generate *Tor^D4021^/CicV* for analysis of maternal effects. *UAS-MEK^F53S^/CyO; CicV/Tm3, sb* and *UAS-MEK^E203K^/CyO; CicV/Tm3, sb* were made by crossing *UAS-MEK^F53S^/CyO; Dr/Tm3,Sb* and *sp/CyO; CicV/CicV*. P(matα-GAL-VP16)mat67; P(matα-GAL-VP16)mat15 (Hunter and Wieschaus, 2000) was used to drive expression of UAS constructs in the early embryo.

### Arraying of embryos for time-lapse microscopy

The device was primed for embryo loading by filling the microchannels with a loading solution of PBST. Air bubbles were removed by closing the device outlet and simultaneously applying ∼10 psi pressure to the device inlet for ∼5 min or until all air bubbles were removed. Embryos were gently delivered through the microfluidic array inlet by applying slight positive pressure to the embryo and PBST suspension. Device loading continued until all or most of the traps were occupied by a single embryo. The fluidic connections at the device inlet and outlet were removed and the arrayed embryos were then imaged.

Time-lapse confocal imaging was done using Zeiss LSM 710 confocal microscope. A Zeiss Plan-Apochromat 20×/0.8 M27 air objective was used for all experiments. A 514 nm argon laser was used to excite CicV fluorescence while 514 nm transmission was used for capturing transmitted light images simultaneously. Embryos were imaged in the AP plane at the embryo midsection with a frequency of 30 s^−1^ for 3 h. The temperature was set to 25°C and maintained via an environmental chamber during imaging.

### Image processing analysis

Pre-processing steps for tissue-level analysis were taken to prepare the raw imaging data for subsequent custom-designed automated analysis. This includes processing raw data in ImageJ and importing in MATLAB for further analysis. Similar to the tissue-level analysis, we used a combination of ImageJ- and MATLAB-based approaches to extract nuclear traces of Cic during nuclear cycle 14 for single-cell analysis.

### Singular value decomposition of Cic dynamics

We applied the singular value decomposition (SVD) to individual movies of Cic dynamics in early embryos, decomposing them into spatial and temporal components (Mardia et al., 1979). All analyses were performed using the Cic signal on the dorsal side of each embryo cross-section. We measured the accuracy of the rank-one approximations by calculating the ratio:(1)
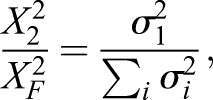


where *X* is the matrix containing the movie of dorsal Cic dynamics, *X*_2_ and *X*_*F*_ are the induced matrix 2 norm and the Frobenius norm of *X*, and *σ*_*i*_ are the ordered singular values of *X* (i.e. *σ*_1_>*σ*_2_>…>*σ*_*n*_, where *n* is the number of time points in the movie). This ratio equals one when the rank of *X* is one and is less than one otherwise. The average values of these ratios for wild type, Tor^D4021^, MEK^F53S^, and MEK^E203K^ were as follows: 0.972±0.003, 0.977±0.002, 0.959±0.009, and 0.968±0.004 (errors are one standard deviation). Each movie was projected onto its respective first spatial mode to produce time-varying amplitudes. We aligned the time courses across embryos by shifting each time axis such that the dip in signal at the 13th nuclear division occurred at the same time in all embryos. With the amplitudes aligned in time, we could proceed with calculating point-wise means and standard deviations of the amplitude across embryos. Additional experimental procedures are in the Supplementary Materials and Methods. They include a description of device fabrication and embryo preparation for loading.
